# Comparative Analysis of Phenolic, Carotenoid, and Elemental Profiles in Three *Crataegus* Species from Şebinkarahisar, Türkiye: Implications for Nutritional Value and Safety

**DOI:** 10.3390/molecules30142934

**Published:** 2025-07-11

**Authors:** Mehmet Emin Şeker, Ayşegül Erdoğan, Emriye Ay

**Affiliations:** 1 School of Tobacco Expertise, Manisa Celal Bayar University, Akhisar 45200, Türkiye; 2 Application and Research Centre for Testing and Analysis (Ege MATAL), Ege University, İzmir 35040, Türkiye; 3 Department of Chemistry, Manisa Celal Bayar University, Yunusemre 45140, Türkiye; emriye.ay@cbu.edu.tr

**Keywords:** *Crataegus*, phenolic compounds, carotenoids, target hazard quotient (THQ), hazard index (HI), carcinogenic risk (CR)

## Abstract

This study evaluated the phenolic, carotenoid, and elemental compositions of three hawthorn species—*Crataegus: C. tanacetifolia* (yellow), *C. orientalis* (orange), and *C. microphylla* (red)—collected from Şebinkarahisar, Türkiye. Liquid chromatography tandem mass spectrometry (LC-MS-MS) analysis revealed that *C. microphylla* had the highest phenolic content, notably epicatechin, gallic acid, and quercetin. It also showed the highest levels of β-carotene and lutein, highlighting its nutraceutical potential. *C. orientalis* was rich in rutin and taxifolin. Inductively coupled plasma mass spectrometry (ICP-MS) results showed significant mineral content, including Fe, Mn, Ca, and Se. About 60 g of dried hawthorn could meet 7–8% of daily selenium needs. In *C. tanacetifolia*, toxicological tests showed no substantial health hazards, with target hazard quotient (THQ) values below 1 and carcinogenic risk (CR) values within tolerable levels (e.g., Ni-CR: 4.68 × 10^−5^). Lead (Pb) and arsenic (As) levels were below detection thresholds in all samples, indicating that hawthorn fruits from this location are safe. The study also shows how species-specific and geographical factors affect hawthorn fruit nutrition and safety.

## 1. Introduction

Hawthorn fruit, known scientifically as *Crataegus*, is a small, hardy fruit that belongs to the *Rosaceae* family [1]. This genus includes over 1000 species widely distributed across Europe, Asia, and North America, and it is adaptable to various climates and soil types [2,3]. The fruit, also called haws or haw berries, has been traditionally used for centuries in folk medicine, particularly for its cardiovascular benefits, where its leaves, blossoms, and berries have been utilized for their anti-inflammatory, antibacterial, and cardioprotective properties [4,5].

The origin of hawthorn is traced back to temperate regions, where it grows naturally in wild, mountainous, and forested areas. In places like Türkiye, hawthorn is especially valued, with different species such as *Crataegus orientalis* and *Crataegus tanacetifolia* found abundantly in rural regions. These species produce fruits that are typically harvested in late autumn and are consumed fresh, dried, or processed into products like jams and teas [3].

Hawthorn fruit is gaining importance not just for its culinary uses but also for its rich medicinal properties [6,7]. It is known for its neuroprotective and cardioprotective effects, which have made it popular in modern pharmaceuticals. Studies have shown that the bioactive compounds in hawthorn, such as flavonoids and phenolic acids, play a crucial role in its health benefits, particularly in reducing blood pressure, improving blood circulation, and providing antioxidant protection against free radicals [8,9,10,11,12]. Among its bioactive compounds, flavonoids and phenolic acids are particularly noteworthy for their antioxidant capacity, playing a key role in protecting against oxidative stress and chronic diseases. This antioxidant property is crucial for preventing chronic diseases such as cardiovascular diseases, certain cancers, and neurodegenerative disorders [13,14,15].

The phenolic composition of hawthorn fruit is particularly significant, as it contributes to its strong antioxidant capacity. It may vary depending on the species and the method of processing, with freeze-dried hawthorn typically showing higher levels of phenolics compared to other drying methods [7,12,16,17,18]. In addition to phenolics, hawthorn fruit is also rich in carotenoids, which are responsible for the red, yellow, or orange pigmentation of the berries [19]. Carotenoids are important for human health due to their role as antioxidants and as precursors to vitamin A, which is vital for vision and immune function. The carotenoid content in hawthorn can also vary based on the species and processing methods, with freeze-drying being more effective in preserving these compounds [20,21,22].

Moreover, hawthorn fruit has been studied for its potential benefits in managing diabetes and improving metabolic health [6,23]. The fruit’s high fiber content, along with its bioactive compounds, contributes to its ability to regulate blood sugar levels and improve lipid profiles. These properties make hawthorn a valuable addition to a health-conscious diet, particularly for individuals at risk of metabolic syndrome [6,7,24]. Given its rich composition of bioactive compounds, hawthorn is not only beneficial for individual health but also holds potential as a natural ingredient in functional foods and nutraceuticals. The increasing interest in natural and plant-based remedies has positioned hawthorn as a promising candidate for further research and development in the fields of nutrition and medicine [25,26].

Mineral content in hawthorn species is of great importance for both health and nutritional reasons [27,28]. The presence of essential minerals such as calcium, potassium, magnesium, and sodium in hawthorn fruits contributes significantly to their nutritional value, supporting various biological functions in the human body. However, the detection and quantification of these minerals, along with PTEs like lead, cadmium, and others, are crucial to ensure the safety of hawthorn consumption [27,29,30]. Studies have shown that hawthorn species can accumulate various minerals in substantial amounts, making it essential to monitor and regulate their levels to prevent potential health risks associated with excessive intake of certain elements [31,32]. This approach not only supports safe consumption but also aids in the selection and cultivation of hawthorn species with favorable nutritional profiles, thus enhancing their value as functional foods and medicinal plants.

This study not only aims to analyze the phenolic compounds, carotenoid content, PTEs, and mineral composition of three hawthorn species from Giresun Şebinkarahisar in Türkiye but also seeks to assess the health implications of these findings through the lens of the target hazard quotient (THQ) and recommended dietary allowance (RDA). By evaluating the PTEs in the hawthorn species and comparing them against the THQ, this research provides critical insights into the potential health risks associated with the consumption of these fruits. Moreover, the mineral content analysis in relation to the RDA offers valuable information on the nutritional benefits and dietary contributions of these hawthorn species. The novelty of this study lies in its dual focus: not only does it present the first detailed biochemical and elemental profile of hawthorn species from this specific region, but it also contextualizes these findings within established health risk and nutritional guidelines, thereby offering a comprehensive evaluation of both the safety and nutritional value of these fruits.

## 2. Results

### 2.1. Composition of Phenolic Compounds

The phenolic compound profile of different *Crataegus* species has attracted considerable interest due to its implications for both health and nutrition. This study conducted a comprehensive analysis of the phenolic content in three species of *Crataegus*, namely *C. tanacetifolia*, *C. orientalis*, and *C. microphylla*. Among these species, *C. microphylla* exhibited the most elevated amounts of various important phenolic compounds, such as epicatechin, quercetin, and vanillin. This is especially remarkable considering the well-established antioxidant qualities of these chemicals, which have been proven to fight against oxidative stress and decrease the likelihood of chronic diseases [33,34]. Based on the findings shown in Table 1, gallic acid was found in *C. microphylla* at a concentration of 305.9 ± 9 μg/g DW. However, it was not detected in *C. tanacetifolia* or *C. orientalis*.

This is significant since gallic acid is a powerful antioxidant that is often present in several fruits, including hawthorn species.

### 2.2. Composition of Carotenoids

The presence of carotenoids in *Crataegus* species demonstrates a wide-ranging and varied distribution of these compounds, which have the capacity to function as antioxidants and contribute to overall well-being. Furthermore, it was discovered that certain *Crataegus* species, including *C. caucasica*, *C. pentagyna*, *C. eriantha*, and *C. curvisepala*, contain carotenoids such as beta-carotene, lutein, and zeaxanthin. Among the previously mentioned species, *C. caucasica* was differentiated due to its wide diversity of carotenoid components, which included beta-carotene, zeaxanthin, cryptoxanthin, and lutein, among others. These dietary ingredients are important because they aid in the prevention of oxidative stress and contribute to the health of the eyes by reducing the risk factors linked with age-related macular degeneration (AMD). The variability in carotenoid levels across several *Crataegus* species indicates their potential as nutraceuticals or natural sources of these crucial compounds. Based on this comprehensive study, it has been shown that *Crataegus* must be taken seriously in terms of carotenoid content [19]. The carotenoid content in different *Crataegus* species shows notable variation (Table 2), with *C. microphylla* exhibiting the highest levels of key carotenoids such as β-carotene, β-cryptoxanthin, and lutein. Specifically, *C. microphylla* has 2.28 mg/g DW of β-carotene, 0.47 mg/g DW of β-cryptoxanthin, and 1.37 mg/g DW of lutein, making it particularly rich in these compounds. These carotenoids are well-known for their antioxidant properties, which contribute to reducing oxidative stress and supporting eye health, thus making *C. microphylla* a valuable species from a nutritional and therapeutic perspective [35,36,37,38,39,40].

### 2.3. Mineral Contents of Crataegus Samples and Recommended Daily Allowance RDA (%) for Adults and Premenopausal Women

The statistical analysis of the microelement and macroelement content in different *Crataegus* species, as presented in Table 3, reveals several significant differences among the species.

For manganese (Mn), the content in *C. tanacetifolia* (5.82 mg/kg) and *C. microphylla* (6.17 mg/kg) is statistically different, as indicated by different letters. However, the Mn content in *C. orientalis* (6.57 mg/kg) does not show a significant difference from either *C. tanacetifolia* or *C. microphylla*, sharing a common letter with both. This suggests that while *C. orientalis* has a slightly higher Mn content, it is not significantly different from the other two species. For iron (Fe), the differences are more pronounced, with *C. orientalis* exhibiting the highest Fe content (42.95 mg/kg), followed by *C. tanacetifolia* (37.87 mg/kg) and *C. microphylla* (23.71 mg/kg). The distinct letters associated with each species indicate statistically significant differences across all three, underscoring the variability in Fe accumulation among the species. In the case of zinc (Zn), *C. microphylla* (10.05 mg/kg) and *C. tanacetifolia* (9.49 mg/kg) show significant differences, as indicated by the different letters. However, the Zn content in *C. orientalis* (8.93 mg/kg) does not differ significantly from the other two species, as it shares a common letter (ab). This pattern suggests some degree of similarity in Zn content between *C. orientalis* and the other species, despite slight variations. Upon evaluation of the analytical findings and the corresponding calculations of daily recommended daily allowance (RDA) values as presented in Table 4, it is apparent that hawthorn (*Crataegus spp.*) fruit is notably rich in essential trace and macroelements such as iron (Fe), manganese (Mn), calcium (Ca), chromium (Cr), copper (Cu), and selenium (Se).

### 2.4. PTE Content of Crataegus Samples

Nickel (Ni) content varies across the species, with *C. tanacetifolia* (1.85 mg/kg), *C. orientalis* (1.12 mg/kg), and *C. microphylla* (1.33 mg/kg) all showing statistically significant differences, as indicated by the letters. In contrast, arsenic (As) content is only significantly lower in *C. microphylla* (0.006 mg/kg) compared to *C. tanacetifolia* and *C. orientalis*, which have identical As content (0.011 mg/kg), as indicated by the shared letter (a) between *C. tanacetifolia* and *C. orientalis*. In all three samples, the concentrations of Pb and As were below the detection threshold. Cr concentrations were recorded as 0.28, 0.46, and 0.91 mg/kg in the species *C. tanacetifolia, C. orientalis*, and *C. microphylla,* respectively. Table 5 shows the concentration of potentially toxic elements.

These results were used to calculate non-carcinogenic and carcinogenic toxicity. Table 5 shows the non-carcinogenic toxicity risk, while Table 6 shows the carcinogenic toxicity risk.

## 3. Discussion

### 3.1. Phenolic Compounds

The literature reveals a paucity of studies analyzing phenolic compounds associated with hawthorn. In the limited number of studies, the presence of flavonoids such as (+)-catechin, (-)-epicatechin, epicatechin gallate, taxifolin, rutin, naringenin, and quercetin as well as phenolic acids such as gallic acid, caffeic acid, caftaric acid, salicylic acid, 4-hydroxybenzoic, and chlorogenic acid was detected [14,34,41,42,43,44].

Upon examining the flavonoid content of the samples, the maximum concentration of epicatechin was found in *C. microphylla* (2479.1 ± 68 μg/g DW), followed by *C. tanacetifolia* (1955 ± 54 μg/g DW), while the lowest concentration was observed in *C. orientalis* (1075 ± 28 μg/g DW). Overall, the literature indicates that these findings align with the epicatechin levels in different *Crataegus* species. For instance, research conducted on *C. monogyna* revealed epicatechin concentrations of approximately 1500 μg/g DW, which are comparatively lower than the levels seen in our study, especially for *C. microphylla* and *C. tanacetifolia*. Additionally, epicatechin concentration was measured as 4.32 mg/g in *Crataegus pubescens* species analyzed in Mexico, although certain species in China exhibited levels as high as 11.72 mg/g [45,46]. These results are quite a bit higher than our findings.

These findings indicate that the *Crataegus* species found in Giresun Şebinkarahisar region may contain elevated levels of epicatechin, potentially due to the unique environmental factors present in the area [44,47]. Quercetin concentrations were highest in *C. microphylla* (88.8 ± 1.9 μg/g DW), followed by lower concentrations in *C. orientalis* (59.1 ± 1.4 μg/g DW) and *C. tanacetifolia* (40.3 ± 1.1 μg/g DW) [44,47]. The findings reported are like those seen in previous research, where the concentration of quercetin in *Crataegus* species varied from 40 to 100 μg/g DW, depending on the specific species and the method used for extraction. The consistency of these values with those in the literature enhances the dependability of the findings in our study and implies that these species exhibit typical levels of quercetin content [8,34].

Taxifolin was detected in significant amounts in *C. orientalis* (516.9 ± 14 μg/g DW) and *C. microphylla* (440.5 ± 11 μg/g DW); however, it was present in much smaller quantities in *C. tanacetifolia* (48.4 ± 0.8 μg/g DW). The levels of taxifolin in *C. orientalis* and *C. microphylla* are higher compared to other *Crataegus* species, which normally have taxifolin content ranging from 200 to 400 μg/g DW. Some findings suggest that some species, especially *C. orientalis*, may possess a particular phytochemical composition characterized by a higher concentration of taxifolin. This could potentially enhance their health-promoting properties [43].

Rutin was found in considerable quantities in *C. orientalis* (351.3 ± 8 μg/g DW) but was significantly lower in *C. microphylla* (13.2 ± 0.3 μg/g DW) and was not discovered in *C. tanacetifolia*. The findings are consistent with the existing literature, which frequently reports increased quantities of rutin in specific *Crataegus* species, especially those recognized for their vascular protective properties. The significant concentration of rutin in *C. orientalis* indicates its potential for utilization in cardiovascular health applications [9,48,49].

When the phenolic acid contents of the samples were examined, according to a study conducted by [27,28], the amount of gallic acid present in various hawthorn species can differ greatly. It has also been observed in other *Crataegus* species like *C. monogyna* at concentrations ranging from 200 to 400 μg/g DW. The specific levels depend on factors such as the extraction method used and the geographical region. The gallic acid level of *C. microphylla* in our study is within the range observed in other hawthorn species documented in the literature, suggesting that this species has similar gallic acid content. *Crataegus microphylla* also stood out for its unique presence of gallic acid (305.9 ± 9 μg/g DW), a compound not detected in *C. tanacetifolia* or *C. orientalis*. This significant difference was underscored by the statistical analysis, which highlighted *C. microphylla* as the sole species containing gallic acid, marking it as particularly rich in this potent antioxidant.

Quercetin, another important flavonoid, also showed significant variability among the species. *C. microphylla* had the highest quercetin content (88.8 ± 1.9 μg/g DW), followed by *C. orientalis* (59.1 ± 1.4 μg/g DW), and *C. tanacetifolia* had the lowest (40.3 ± 1.1 μg/g DW). The statistical analysis confirmed that the differences in quercetin levels across these species were significant, with different letters assigned to each value, reflecting the substantial variation in quercetin content

Vanillin was detected in *C. microphylla* (110.1 ± 2.9 μg/g DW) and *C. orientalis* (106.9 ± 3.1 μg/g DW) at comparable concentrations, which were greater than those found in *C. tanacetifolia* (66.2 ± 1.3 μg/g DW). To the best of our knowledge, vanillin is typically an important phenolic compound; however, there is no reported value in any *Crataegus* species so far. *C. microphylla* and *C. orientalis* had similarly high levels (110.1 ± 2.9 μg/g DW and 106.9 ± 3.1 μg/g DW, respectively), both significantly higher than *C. tanacetifolia* (66.2 ± 1.3 μg/g DW). This similarity between *C. microphylla* and *C. orientalis* and their significant difference from *C. tanacetifolia* was highlighted by the statistical analysis, which grouped the two higher concentrations under the same letter, distinct from the lower concentration in *C. tanacetifolia*.

Protocatechuic aldehyde was detected in *C. tanacetifolia* at a concentration of 0.61 ± 0.022 μg/g DW and in *C. orientalis* at a concentration of 4.02 ± 0.03 μg/g DW. However, it was not found in *C. microphylla*. This chemical has been previously detected in hawthorn species in previous studies, where it has been found in little amounts, usually less than 5 μg/g DW. The amounts observed in *C. orientalis* correlate with the higher end of the values reported in the literature, suggesting that *C. orientalis* may have a little greater ability to produce this chemical compared to other species [8,34,50].

Caffeic acid was detected in all three species, with *C. tanacetifolia* exhibiting the greatest concentration (15.3 ± 0.4 μg/g DW), followed by *C. orientalis* (10.3 ± 0.22 μg/g DW) and *C. microphylla* (6.6 ± 0.05 μg/g DW). The values fall within the reported range found in other studies, where the amount of caffeic acid in hawthorn species typically ranges from 5 to 20 μg/g DW. The findings indicate that *C. tanacetifolia* may possess a significantly greater ability to produce caffeic acid in comparison to other hawthorn species. This could potentially contribute to its antioxidant characteristics [8,43].

The presence of p-coumaric acid was observed exclusively in *C. microphylla* at a concentration of 33.3 ± 0.8 μg/g DW. This chemical is frequently found in trace amounts in hawthorn species, with reported levels typically ranging from 10 to 30 μg/g DW. The level observed in *C. microphylla* is higher than the usual range, indicating a potentially greater antioxidant capacity in this species relative to others [34,43].

The species exhibited significant variation in salicylic acid content, with *C. orientalis* having the highest concentration (10.1 ± 0.2 μg/g DW), followed by *C. tanacetifolia* (2.6 ± 0.03 μg/g DW) and *C. microphylla* with the lowest concentration (1.4 ± 0.04 μg/g DW). The occurrence of salicylic acid in these species, especially in *C. orientalis*, corresponds to its established anti-inflammatory characteristics, as evidenced in previous research on *Crataegus* species [34].

4-Hydroxy benzoic acid was detected in all three species, with the highest concentration found in *C. orientalis* (11.6 ± 0.18 μg/g DW), followed by *C. microphylla* (3.2 ± 0.09 μg/g DW) and *C. tanacetifolia* (2.8 ± 0.03 μg/g DW). The observed findings match the information found in literature, which suggests that levels of 4-OH benzoic acid in hawthorn species typically fall between the range of 2 to 12 μg/g DW. This indicates that *C. orientalis* might have a somewhat greater antioxidant potential due to its higher concentration of this component [41,44].

4-Hydroxybenzoic acid content varied significantly, with *C. orientalis* showing the highest level (11.6 ± 0.18 μg/g DW), significantly higher than both *C. microphylla* (3.2 ± 0.09 μg/g DW) and *C. tanacetifolia* (2.8 ± 0.03 μg/g DW). The post hoc analysis confirmed these differences, grouping *C. orientalis* separately from the other two species, which had relatively similar but lower levels of this compound.

The comparison demonstrates that the phenolic profiles of the *Crataegus* species examined in our study are in line with, and in certain cases exceed, those stated in the literature. The elevated concentrations of specific phenolic compounds, particularly in *C. microphylla* and *C. orientalis*, indicate that these species might possess increased health advantages, which justifies the need for additional investigation and their utilization in functional foods or nutraceuticals.

### 3.2. Carotenoids

The variability in carotenoid levels across several *Crataegus* species indicates their potential as nutraceuticals or natural sources of these crucial compounds. Based on this comprehensive study, it has been shown that *Crataegus* must be taken seriously in terms of carotenoid content [19]. The carotenoid content analysis also showed significant differences among the three species. For β-carotene, *C. microphylla* had the highest content (2.28 ± 0.04 mg/g DW), significantly higher than *C. orientalis* (1.69 ± 0.04 mg/g DW) and *C. tanacetifolia* (1.36 ± 0.05 mg/g DW). The statistical analysis clearly differentiated these species based on their β-carotene content, which is crucial for their provitamin A activity. Lutein content followed a similar pattern, with *C. microphylla* having the highest level (1.37 ± 0.05 mg/g DW), followed by *C. orientalis* (0.91 ± 0.07 mg/g DW) and *C. tanacetifolia* (0.48 ± 0.04 mg/g DW). These differences were statistically significant, as indicated by the different letters in the table, confirming the superior lutein content in *C. microphylla*. In contrast, β-cryptoxanthin content showed a different trend, where *C. orientalis* and *C. tanacetifolia* had similar levels (0.56 ± 0.04 mg/g DW and 0.51 ± 0.06 mg/g DW, respectively), while *C. microphylla* had a slightly lower content (0.47 ± 0.02 mg/g DW). The statistical analysis indicated that the differences between *C. orientalis* and *C. tanacetifolia* were not significant, as they were grouped under the same letter, but *C. microphylla* was significantly different from *C. orientalis*. Overall, the statistical results highlight the significant biochemical diversity among the three *Crataegus* species, with each species demonstrating unique profiles in terms of both phenolic and carotenoid compounds. These differences have important implications for the nutritional and medicinal applications of these species.

### 3.3. Mineral Contents and Recommended Daily Allowance RDA (%)

The statistical analysis of the microelement and macroelement content in different *Crataegus* species, as presented in Table 4, reveals several significant differences among the species. For manganese (Mn), the content in *C. tanacetifolia* (5.82 mg/kg) and *C. microphylla* (6.17 mg/kg) is statistically different, as indicated by different letters. However, the Mn content in *C. orientalis* (6.57 mg/kg) does not show a significant difference from either *C. tanacetifolia* or *C. microphylla*, sharing a common letter with both. This suggests that while *C. orientalis* has a slightly higher Mn content, it is not significantly different from the other two species. For iron (Fe), the differences are more pronounced, with *C. orientalis* exhibiting the highest Fe content (42.95 mg/kg), followed by *C. tanacetifolia* (37.87 mg/kg) and *C. microphylla* (23.71 mg/kg). The distinct letters associated with each species indicate statistically significant differences across all three, underscoring the variability in Fe accumulation among the species. In the case of zinc (Zn), *C. microphylla* (10.05 mg/kg) and *C. tanacetifolia* (9.49 mg/kg) show significant differences, as indicated by the different letters. However, the Zn content in *C. orientalis* (8.93 mg/kg) does not differ significantly from the other two species, as it shares a common letter (ab). This pattern suggests some degree of similarity in Zn content between *C. orientalis* and the other species despite slight variations. For example, while a 30 g portion of chocolate [51] or hazelnuts [52] provide approximately 3–4% of the daily selenium requirement, the consumption of 60 g of dried hawthorn fruit supplies around 7–8% of the recommended daily intake for this element. These findings suggest that hawthorn fruit contains selenium at levels comparable to or even exceeding those found in commonly consumed selenium sources such as chocolate and hazelnuts. It is important to note that these values are calculated based on dried fruit samples, as drying was performed prior to the analytical procedures. Therefore, when considering the nutritional contribution of fresh hawthorn fruit, the elemental concentrations should be adjusted accordingly, typically resulting in a reduction of approximately 90%, given the moisture content of fresh fruit.

A comparative study conducted by Özcan et al., 2005 that investigated the elemental composition of hawthorn fruits collected from the Konya region reported values that deviate substantially from those obtained in the present study [32]. While the concentrations of Fe and Ca were relatively consistent across both studies, significantly higher levels of magnesium (Mg) and sodium (Na) were reported in the Konya samples. In contrast, the Şebinkarahisar samples analyzed in the current study exhibited markedly elevated concentrations of potassium (K) and selenium (Se), indicating a potential geographical influence on elemental accumulation.

### 3.4. Evaluation of THQ and CR Values in Crataegus Samples

The evaluation of PTEs in *Crataegus* species is essential for ensuring their safety as food and medicinal sources, as these elements can pose serious health risks when present above permissible levels. Table 6 shows the content of some PTEs in *Crataegus* samples. Based on the results, a notable difference was observed in lead (Pb) content: while the Konya samples contained Pb at a concentration of 0.71 ppm, it was below the detection limit in the Şebinkarahisar samples. These findings collectively underscore the significant impact of geographical and environmental factors on the elemental composition of hawthorn fruits and highlight the importance of regional assessments for accurate nutritional and toxicological evaluations. The variation in concentrations of the three distinct species taken from the same place implies that the results may be associated with the chemical composition of the species. Given that Şebinkarahisar is a high-altitude district and remote from urbanization, it is expected that the concentrations of Pb and As were below the detection limit.

The evaluation of non-carcinogenic toxicity (THQ) revealed that all three samples have findings of less than 1, indicating no associated risk. When evaluating CR, findings below 10^−6^ are deemed non-risky. Results ranging from 10^−4^ to 10^−6^ are deemed intermediate risk but permissible. Results over 10^−4^ are deemed hazardous [53,54,55]. Table 5 presents the THQ values of PTEs in dried *Crataegus* samples. All THQ values for the elements analyzed were found to be well below the critical threshold of 1. This indicates that consumption of these fruits does not pose a significant non-carcinogenic health risk to the general population. Notably, the highest individual THQ value observed was 9.00 × 10^−3^, which remains nearly two orders of magnitude below the risk threshold. These findings confirm the toxicological safety of the hawthorn species studied under typical consumption levels. Table 6 details the estimated CR associated with exposure to specific toxic metals (Cr, Pb, Cd, Ni, and As) for both male and female consumers. All CR values fall within the range of 1.0 × 10^−6^ to 1.0 × 10^−4^, which is generally considered the acceptable or tolerable risk range according to USEPA guidelines. Among the metals, nickel (Ni) presented the highest CR values across all samples, particularly in *C. tanacetifolia* for females (4.8 × 10^−5^) and in *C. microphylla* (3.4 × 10^−5^), indicating a slightly elevated but still acceptable level of risk. Other elements such as lead (Pb) and cadmium (Cd) showed CR values as low as ~10^−10^ and ~10^−9^, respectively, demonstrating negligible carcinogenic potential in the tested samples. Overall, both the THQ and CR data suggest that the consumption of dried *Crataegus* fruits from the Şebinkarahisar region does not pose significant health hazards in terms of toxic metal exposure, thereby supporting their safety for dietary and nutraceutical use. However, the relatively higher CR values for Ni warrant continued monitoring and regional comparison, especially if hawthorn-based products are to be consumed regularly or in higher quantities.

## 4. Materials and Methods

### 4.1. Chemicals and Reagents

Catechin, taxifolin, epicatechin, gallic acid, caffeic acid, ferulic acid, ellagic acid, protocatechuic acid, protocatechuic aldehyde, vanillin, quercetin, kaempferol, p-coumaric acid, resveratrol, salicylic acid, syringic acid, sesamol, rutin, 4-hydroxybenzoic acid, rosmarinic acid, flavone, and oleuropein standards were purchased from Merck (Darmstadt, Germany) from Sigma Aldrich (St. Louis, MO, USA). All-trans-lutein, all-trans beta carotene, and all-trans beta cryptoxanthin were provided form CaroteNature (Lupsingen, Switzerland). Triethylamine, potassium hydroxide, sodium sulphate, calcium chloride, and calcium carbonate were provided by Sigma-Aldrich (Darmstadt, Germany), and all the LC-grade solvents and ACS-grade ethanol used in this study were purchased from Merck (Darmstadt, Germany).

### 4.2. Collection of Crataegus Samples

Hawthorn (*Crataegus* spp.) fruits representing three distinct species, each with a characteristic fruit color, were systematically collected from the Şebinkarahisar district of Giresun Province, located in the eastern Black Sea region of Türkiye in October 2022. The selection and collection of samples were carried out during the optimal ripening period, ensuring the morphological integrity and phytochemical representativeness of each species. The yellow-fruited samples were identified as *Crataegus tanacetifolia*, a species commonly found in rocky and mountainous terrains. The orange-fruited samples belonged to *Crataegus orientalis*, which is widespread across Anatolia and known for its moderate altitude adaptability. Lastly, the red-fruited specimens were determined to be *Crataegus microphylla*, typically observed in forested or shrubby areas at higher elevations. Each sample group was collected from naturally growing populations under similar ecological conditions to minimize environmental variability. The fruits were then labeled, transported under controlled conditions, and stored at –20 °C. They were washed with tap water and rinsed with deionized water to remove impurities. After drying on blotting paper, they were oven-dried at 40 °C to constant weight. The dried samples were ground into a fine powder prior to analysis.

### 4.3. Extraction of Phenolic Compounds

Phenolic compounds were extracted from the hawthorn (*Crataegus* spp.) fruit samples using the Soxhlet extraction method, a conventional yet effective solid–liquid extraction technique. To enhance the selectivity for phenolic compounds, a polar solvent system methanol was employed, which favors the dissolution of polyphenols while minimizing the co-extraction of non-polar compounds such as fats. Additionally, prior to analysis, samples were centrifuged and filtered to eliminate particulate and insoluble matter, helping to reduce interference from proteins and sugars. Initially, 5.0 g of each dried and homogenized fruit sample were accurately weighed and finely pulverized using a laboratory blender to increase the surface area and enhance solvent penetration. The pulverized material was then carefully transferred into a cellulose extraction thimble and placed inside the Soxhlet extraction chamber. A volume of 150 mL of high-purity methanol (analytical grade) was used as the extraction solvent due to its polarity and efficacy in dissolving phenolic constituents. The Soxhlet extraction was conducted for a continuous period of 6 h, allowing for repeated solvent cycling to ensure maximal compound recovery. Upon completion of the extraction, the methanolic extract was subjected to filtration using Whatman No.1 filter paper to eliminate any particulate residues. The filtrate was subsequently concentrated using a rotary evaporator, (Heidolph, Heidolph Instruments GmbH & Co. KG, Schwabach, Germany) under reduced pressure at 40 °C and 175 mbar to gently remove the methanol without degrading thermolabile phenolic compounds. The resulting concentrated extracts were then reconstituted in a methanol–water solution (25:75, *v*/*v*) to enhance the solubility and stability of the phenolics. These prepared samples were stored at –20 °C in amber vials until further analysis by liquid chromatography coupled with tandem mass spectrometry (LC-MS/MS), which provides sensitive and accurate quantification of individual phenolic constituents.

### 4.4. Extraction of Carotenoids

In accordance with the validated protocol described by Erdoğan et al., 2015 [56], carotenoids were extracted from *Crataegus* fruit samples using an ultrasound-assisted extraction (UAE) technique, followed by saponification. For each extraction, precisely 1 g of the homogenized *Crataegus* sample was weighed and combined with 1 g of calcium carbonate (CaCO_3_), which serves to neutralize organic acids and prevent carotenoid degradation during the extraction process. Ethanol was selected as the extraction solvent due to its polarity, food-grade safety, and efficacy in solubilizing carotenoids. The ultrasound-assisted extraction was carried out using an Elmasonic S80H, (Elma Schmidbauer GmbH, Singen, Germany) ultrasonic bath, which promotes efficient cell disruption and enhances the release of intracellular carotenoids through acoustic cavitation.

Following extraction, the samples were subjected to a saponification step to remove chlorophylls, lipids, and other interfering compounds. This process involved the addition of 10% methanolic potassium hydroxide (KOH) solution, and the reaction was maintained for 2 h at room temperature, as specified in the validated methodology. The saponified mixture was then treated to isolate the ether phase, which contains the liberated carotenoids. To prepare the extract for LC-MS/MS analysis, 10 mL of ethanol was added to dissolve the ether phase completely, ensuring the full recovery of carotenoids in a form suitable for accurate chromatographic quantification and identification.

### 4.5. Validation of Extraction Methods

To the best of current scientific knowledge, there is no single standard reference material (SRM) available that universally corresponds to every type of matrix material for method validation purposes. This limitation necessitates the selection of SRMs with matrices that closely resemble the target sample, thereby serving as representative reference materials to ensure method reliability and accuracy. In this study, SRM 3246 Ginkgo biloba (leaves) was employed for the validation of the phenolic compound extraction procedure. Although not identical to the target sample, this SRM was considered appropriate due to its classification as a food matrix and its compositional similarity to plant-based materials rich in phenolic constituents. The validation results obtained using this material are summarized in Table 1, demonstrating the applicability and robustness of the proposed extraction method. Likewise, for the validation of the carotenoid extraction, BCR 485 mixed vegetables—a certified reference material widely used in previous studies—was selected as a representative food matrix. Its complex vegetable composition, which naturally contains a diverse range of carotenoids, renders it suitable for assessing the efficiency and accuracy of carotenoid extraction protocols [57].

### 4.6. Preparation of Samples for ICP-MS Measurements

The vegetable samples were initially washed at least three times under running tap water to eliminate surface impurities and particulate matter. Subsequently, they were rinsed thoroughly with deionized water. The cleaned samples were then placed on blotting paper to remove residual surface moisture, followed by oven-drying at 80 °C until constant weight was achieved, ensuring complete removal of water content. Prior to analysis, all dried vegetable samples were ground into a fine powder using a laboratory-grade grinder as applied by other researchers [58]. To decompose the dehydrated and ground *Crataegus* samples and release the elements into solution, a closed-vessel high-pressure microwave-assisted wet digestion (MWD) technique was utilized. Each sample, weighing precisely 0.25 g, was placed in Teflon tubes, and 2.0 mL of a mixture of nitric acid (HNO_3_) and hydrochloric acid (HCl) in a 5:2 volume ratio was added [52,59,60]. The digestion process was carried out using a microwave digestion oven (CEM Mars 5) set to 1600 W and 210 °C for 20 min. Following digestion, the vessels were allowed to cool, and the solutions were vortexed. Deionized water was then added to adjust the total volume to 10 mL prior to ICP-MS analysis. Each experiment was repeated three times, and the relative standard deviations were calculated for each sample. This method was validated within the scope of the study conducted by Erdoğan et al., 2024 [51].

### 4.7. ICP-MS Analysis of Elements

A total of 14 elements, comprising both essential minerals and potentially toxic elements (Mn, Fe, Zn, Se, Cr, Cu, Mg, Ca, Na, K, Pb, Cd, Ni, and As), were quantified in three different samples using an inductively coupled plasma mass spectrometry (ICP-MS) system (Agilent Technologies 7700X, Agilent Technologies Inc., Santa Clara, CA, USA). To correct for potential matrix effects, internal standards including ^45^Sc, ^89^Y, ^103^Rh, ^115^In, and ^209^Bi were employed throughout the analysis. The ICP-MS operating conditions were as follows: plasma gas flow rate of 15.00 L/min, auxiliary gas flow rate of 1.50 L/min, sheath gas flow rate of 0.200 L/min, nebulizer gas flow rate of 0.960 L/min, sampling depth of 7.00 mm, and radio frequency power of 1.40 kW.

Prior to sample analysis, calibration curves were prepared for each element using eight concentration levels (0.001, 0.002, 0.005, 0.100, 0.200, 0.500, and 1.00 mg/L). Each concentration level was measured in quadruplicate, and the mean values were used to construct the calibration curves. Final elemental concentrations in the samples were calculated by applying appropriate dilution factors to the values obtained from the calibration plots.

### 4.8. LC-MS/MS Analyses of Carotenoids and Phenolic Compounds

Carotenoid analysis in this study was performed using liquid chromatography tandem mass spectrometry (LC-MS/MS) equipped with an Atmospheric Pressure Chemical Ionization (APCI) source based on the methodology applied in previous studies [61,62]. The mass spectrometer (Thermo Scientific TSQ Quantum Access Max, Thermo Fisher Scientific Inc., Waltham, MA, USA) operated in full scan mode over an *m*/*z* range of 50–900, with a vaporization temperature set at 350 °C. Separation was achieved using a YMC C_30_ column (4.6 × 250 mm, 5 µm) under a gradient elution program. The initial mobile phase consisted of 70% methanol, 5% water (with 0.1% formic acid), and 25% methyl-tert-butyl ether. At 5 min, the composition was modified to 60% methanol and 35% methyl-tert-butyl ether; by 10 min, it shifted to 45% methanol and 55% methyl-tert-butyl ether. At the end of the 15 min run, the mobile phase was adjusted to 25% methanol and 75% methyl-tert-butyl ether. Identification and retention times of lutein and β-carotene were determined using analytical standards. The mass spectrometric analysis was performed in positive ion mode, optimized using commercial standards of lutein and β-carotene. Selected ion monitoring (SIM) was applied, using *m*/*z* 569, 551, and 459 for lutein and *m*/*z* 537, 445, and 431 for β-carotene.

The quantification of phenolic compounds including gallic acid, protocatechuic aldehyde, sesamol, epicatechin, caffeic acid, vanillin, taxifolin, p-coumaric acid, 4-hydroxybenzoic acid, salicylic acid, rutin, and quercetin was performed using LC-MS/MS based on external standard calibration with authentic reference standards. Phenolic compounds were separated using a C_18_ column (ODS Hypersil, 4.6 mm × 250 mm, 5 µm) maintained at 30 °C, with a flow rate of 0.7 mL/min. The injection volume was 20 µL, and the total analysis time was 30 min. The mobile phases consisted of solution A (0.1% formic acid in water) and solution B (methanol). The elution program began with 100% A for the first minute. From 1 to 22 min, a linear gradient was applied to reach 5% A and 95% B. This composition (5% A–95%min B) was maintained for an additional 3 min. Subsequently, from 25 to 30 min, the system was adjusted to 100% B (0% A). The system was shut down after 30 min. Instrumental conditions were set as follows: capillary temperature at 300 °C, vaporizer temperature at 350 °C, spray voltage of 4000 V in positive mode, and 2500 V in negative mode. This method was previously employed in previous studies for the separation and quantification of phenolic compounds [63].

### 4.9. Assessment of Health Risks

A comprehensive health risk assessment was conducted to evaluate the potential risks associated with the presence of selected trace elements in the *Crataegus* fruit samples. The elements assessed included manganese (Mn), iron (Fe), nickel (Ni), copper (Cu), zinc (Zn), cadmium (Cd), lead (Pb), arsenic (As), and chromium (Cr), which encompass both essential micronutrients and potentially toxic elements. To quantify the potential human health impacts, several risk assessment parameters were calculated: estimated daily intake (EDI), target hazard quotient (THQ), hazard index (HI), and target cancer risk (CR). These metrics collectively offer insight into both non-carcinogenic and carcinogenic risks posed by dietary exposure to the analyzed elements. The tolerable daily intake (TDI) refers to the maximum amount of a substance—whether present in food, water, air, or other environmental media—that can be ingested daily over a lifetime without posing appreciable health risks. This threshold is determined by regulatory authorities such as the European Food Safety Authority (EFSA), as referenced [53].

In the present study, the average daily fruit consumption for individuals in the studied region was set at 30 g per day, based on local dietary habits and available consumption data. This value was used as a foundational input for estimating daily exposure levels and comparing them against established safety thresholds. Consequently, the EDI in milligrams per kilogram of body weight per day was computed utilizing Formula (1):

(1) $$\mathrm{EDI}=\;\frac{C\;\times \;\mathrm{Cf}\;\times \;\mathrm{DC}}{\mathrm{BW}}{\;\times \;10}^{-3}$$

Cf denotes the daily average fruit consumption (5000 mg/day), C signifies the metal concentration in fruit (mg/kg), Cf is the conversion factor (0.085), and BW indicates the typical adult body weight (70 kg) for men and (60 kg) for women [54].

The THQ was computed to evaluate the non-carcinogenic health concerns linked to contaminants. The THQ is the ratio of the EDI of a pollutant to the reference dosage (RfD) established by regulatory authorities. A THQ score exceeding one signifies an elevated risk of negative health consequences from exposure to that pollutant. Consequently, the THQ was computed to evaluate the non-carcinogenic risk. The THQ was computed to evaluate the risk of ingesting metal contaminants through fruits and to highlight the potential dangers linked to the habitual ingestion of these contaminants.

The computation was executed utilizing Formula (2):

(2) $$THQ=\frac{EF\times ED\times IRd\times MC}{RfD\times BW\times {AT}_{noncancer}}\times 10^{-3}$$

In this context, EF denotes the exposure frequency, generally established at 350 days annually. ED signifies the exposure duration, typically set at 26 years for non-cancer risk evaluations, in accordance with the methodology employed by the United States Environmental Protection Agency [55]. However, since the fruit is collected and eaten seasonally, the total consumption period in this study was taken as 3 months per year. BW represents the mean adult body weight in kilograms, typically standardized to 70 kg for calculating purposes. AT denotes the average duration for non-carcinogenic agents, computed as 365 days annually multiplied by an exposure duration of 50 years.

RfD denotes the oral reference dose, a metric utilized to evaluate the potential health hazards linked to exposure to a particular chemical. A hazard index (HI) was computed by summing the (THQ) values of all contaminants present in the fruit samples. The HI evaluates the overall non-carcinogenic health hazards linked to the consumption of spoilt fruit. An HI value exceeding one signifies a potential health risk due to the cumulative impact of several contaminants. Consequently, the computation of the HI was executed utilizing Formula (3).

(3) $$HI=\sum_{i=1}^{n}{THQ}_{i}$$

Target cancer risk serves as a critical metric in assessing the likelihood of carcinogenic effects resulting from exposure, thereby informing risk management strategies and regulatory decisions. Calculating CR typically considers factors such as exposure frequency, duration, carcinogen potency, and individual susceptibility. This comprehensive approach enables a nuanced understanding of the potential CR associated with different environmental exposures, thereby facilitating informed decision making to protect public health. The concept of carcinogenic hazards has been illustrated through the measurement of CR, which is quantified using a specific formula denoted as Formula (4).

(4) $$CR=\frac{EF\times ED\times IRd\times MC\times CSF}{BW\times {AT}_{cancer}}\times 10^{-3}$$

The CSF values, in the unit of mg/kg/day, from the Integrated Risk Information System for oral carcinogenic slope factors are Pb = 0.0085, Cd = 0.38, Ni = 1.7, and Cr = 0.5 (mg kg^−1^ day)^−1^ [52].

### 4.10. Statistical Analysis

In the present study, comprehensive statistical analyses were conducted from multiple methodological perspectives to evaluate the variations among the *Crataegus* samples based on specific analytical parameters. All analyses were performed in triplicate, and results are expressed as mean values. The dataset was subjected to one-way analysis of variance (ANOVA) using Minitab statistical software (Version 18, Minitab Inc., State College, PA, USA) to determine whether there were statistically significant differences among the *Crataegus* samples for the measured variables.

Following the identification of significant effects through ANOVA, Tukey’s test was applied as a post hoc procedure to perform pairwise comparisons among group means. Descriptive statistics, including mean values and standard deviations (SD), were calculated to provide a comprehensive overview of the data distribution. A significance level of *p* < 0.05 was adopted throughout the analysis to assess the statistical relevance of the observed differences.

## 5. Conclusions

This study provides a comprehensive comparative analysis of the phenolic, carotenoid, and elemental composition of three hawthorn (*Crataegus*) species—*C. tanacetifolia*, *C. orientalis*, and *C. microphylla*—collected from Şebinkarahisar, Türkiye. The results reveal significant biochemical diversity among the species, particularly in terms of phenolic profiles, carotenoid concentrations, and essential mineral content. *C. microphylla* emerged as the most promising species due to its superior levels of key bioactive compounds such as epicatechin, quercetin, β-carotene, and lutein, which are associated with antioxidant and cardioprotective effects. In terms of mineral composition, all three species were found to be rich sources of essential elements such as Fe, Mn, Ca, Cr, Cu, and Se. The calculated dietary intake values demonstrated that dried hawthorn fruit can contribute substantially to the recommended daily intake of these elements, especially selenium. However, it is critical to consider the moisture content of fresh fruits when translating these findings into dietary recommendations. Furthermore, the assessment of PTEs, alongside health risk indicators such as THQ and CR, confirmed that all three species are safe for human consumption, with no significant non-carcinogenic or carcinogenic risks identified under typical consumption scenarios. The absence of lead in Şebinkarahisar samples and regional differences in elemental profiles underscore the influence of geographical and environmental factors on the nutritional quality and safety of hawthorn fruits. Overall, this study highlights the nutritional and functional potential of hawthorn fruits, particularly *C. microphylla*, as valuable candidates for use in functional food formulations and nutraceutical development.

## Figures and Tables

**Table 1:** Results of phenolic compounds (μg/g DW) in different *Crataegus* samples.

Phenolic Compounds	*C. tanacetifolia*	*C. orientalis*	*C. microphylla*
**Flavonoids**			
Rutin	ND	351.3 ± 8 ^a^	13.2 ± 0.3 ^b^
Sesamol	ND	3.64 ± 0.02 ^a^	ND
Epicatechin	1955 ± 54 ^a^	1075 ± 28 ^b^	2479.1 ± 68 ^c^
Caffeic Acid	15.3 ± 0.4 ^a^	10.3 ± 0.22 ^b^	6.6 ± 0.05 ^c^
Vanillin	66.2 ± 1.3 ^a^	106.9 ± 3.1 ^b^	110.1 ± 2.9 ^b^
Taxifolin	48.4 ± 0.8 ^a^	516.9 ± 14 ^b^	440.5 ± 11 ^c^
Quercetin	40.3 ± 1.1 ^a^	59.1 ± 1.4 ^b^	88.8 ± 1.9 ^c^
**Phenolic Acids**			
p-Coumaric Acid	ND	ND	33.3 ± 0.8 ^a^
4-Hydroxybenzoic Acid	2.8 ± 0.03 ^a^	11.6 ± 0.18 ^b^	3.2 ± 0.09 ^c^
Salicylic Acid	2.6 ± 0.03 ^a^	10.1 ± 0.2 ^b^	1.4 ± 0.04 ^c^
Gallic Acid	ND	ND	305.9 ± 9 ^a^
**Aldehydic Phenols**			
Protocatechualdehyde	40.3 ± 1.1 ^a^	59.1 ± 1.4 ^b^	88.8 ± 1.9 ^c^

Different letters (“a”, “b” and “c”) on the same line indicate a significant difference according to the post hoc Tukey’s test (*p* < 0.05). The results from three analytical replicates were calculated, and the data for each experiment were mean values. ND, not detected.

**Table 2:** Carotenoid content (mg/g DW) of different *Crataegus* samples.

Carotenoids	*C. tanacetifolia*	*C. orientalis*	*C. microphylla*
β-Carotene	1.36 ± 0.05 ^a^	1.69 ± 0.04 ^b^	2.28 ± 0.04 ^c^
β-Cryptoxanthin	0.51 ± 0.06 ^a^	0.47 ± 0.02 ^b^	0.56 ± 0.04 ^a^
Lutein	1.37 ± 0.05 ^c^	0.91 ± 0.07 ^b^	0.48 ± 0.04 ^a^

Different letters (“a”, “b” and “c”) on the same line indicate a significant difference according to the post hoc Tukey’s test (*p* < 0.05). The results from three analytical replicates were calculated, and the data for each experiment were mean values.

**Table 3:** Content (mg kg^−1^ DW) of microelements, macroelements, and PTEs determined in the *Crataegus* samples.

Elements	*C. tanacetifolia*	*C. orientalis*	*C. microphylla*
Mn	5.82 ± 0.18 ^a^	6.57 ± 0.26 ^a,b^	6.17 ± 0.15 ^b^
Fe	37.87 ± 1.15 ^a^	42.95 ± 1.72 ^b^	23.71 ± 0.59 ^c^
Zn	9.49 ± 0.29 ^a^	8.93 ± 0.357 ^a,b^	10.05 ± 0.25 ^b^
Se	0.07 ± 0.002 ^a^	0.07 ± 0.003 ^a^	0.06 ± 0.002 ^b^
Cr	0.28 ± 0.0085 ^a^	0.46 ± 0.0184 ^b^	0.91 ± 0.0228 ^c^
Cu	6.86 ± 0.21 ^a^	4.95 ± 0.19 ^b^	4.36 ± 0.11 ^c^
Mg	495.7 ± 8.2 ^a^	481.5 ± 9.3 ^a^	483.1 ± 7.1 ^a^
Ca	2745 ± 61.6. ^a^	2959 ± 81.4 ^a^	3722 ± 93.2 ^b^
Na	57.1 ± 1.74 ^a^	53.2 ± 2.12 ^a,b^	58.4 ± 1.45 ^b^
K	6000.1 ± 18.3 ^a^	5557.0 ± 22.2 ^b^	7367.2 ± 18.4 ^b^
Pb	<0.001	<0.001	<0.001
Cd	<0.001	<0.001	<0.001
Ni	1.85 ± 0.037 ^a^	1.12 ± 0.022 ^b^	1.33 ± 0.027 ^c^
As	0.011 ± 0.00022 ^a^	0.011 ± 0.00020 ^a^	0.006 ± 0.00012 ^b^

Different letters (“a”, “b” and “c”) in the same row are statistically significant (*p* < 0.05) according to the post hoc Tukey’s test. The results from three analytical replicates were calculated, and the data for each experiment were mean values.

**Table 4:** Recommended daily allowance RDA (%) values of *Crataegus* samples.

Mineral Elements	RDA/Al/UL	*C. tanacetifolia*	*C. orientalis*	*C. microphylla*
Mn	2.3 mg	15.2	17.1	16.1
	1.8 mg	19.4	21.9	20.6
Fe	8 mg	28.4	32.2	17.8
	18 mg	12.6	14.3	7.9
Zn	11 mg	5.2	4.9	5.5
	8 mg	7.1	6.7	7.5
Mg	420 mg	7.1	6.9	6.9
	320 mg	9.3	9	9.1
Cr	0.035 mg	47.8	77.8	156.2
	0.025 mg	66.9	108.9	218.6
Ca	1000 mg	16.5	17.8	22.3
Na	1500 mg	0.02	0.02	0.02
K	4700 mg	7.7	7.1	9.4
Se	0.055 mg	8.4	8.6	7.3
Cu	0.9 mg	45.7	33	29.1
Ni	1 mg	11.1	6.7	8

**Table 5:** Target hazard quotient (THQ) values of PTEs in *Crataegus* samples.

Samples	Gender	Mn	Fe	Zn	Cr	Cu	Pb	Cd	Ni	As	HI
*C. tanacetifolia*	M	5.40 × 10^−4^	7.00 × 10^−4^	4.10 × 10^−4^	1.00 × 10^−3^	2.20 × 10^−3^	2.30 × 10^−6^	1.30 × 10^−4^	9.00 × 10^−4^	4.70 × 10^−4^	6.40 × 10^−3^
	FM	6.30 × 10^−4^	1.20 × 10^−3^	4.80 × 10^−4^	1.20 × 10^−3^	2.60 × 10^−3^	3.79 × 10^−6^	1.50 × 10^−4^	1.40 × 10^−3^	5.50 × 10^−4^	8.20 × 10^−3^
*C. orientalis*	M	6.10 × 10^−4^	8.00 × 10^−4^	3.90 × 10^−4^	1.70 × 10^−3^	1.60 × 10^−3^	2.3 × 10^−6^	1.30 × 10^−4^	5.50 × 10^−4^	4.70 × 10^−4^	6.20 × 10^−3^
	FM	7.10 × 10^−4^	1.30 × 10^−3^	4.50 × 10^−4^	2.00 × 10^−3^	1.90 × 10^−3^	3.79 × 10^−6^	1.50 × 10^−4^	8.50 × 10^−4^	5.50 × 10^−4^	7.90 × 10^−3^
*C. microphylla*	M	5.70 × 10^−4^	4.40 × 10^−4^	4.40 × 10^−4^	3.30 × 10^−3^	1.40 × 10^−3^	2.3 × 10^−6^	1.30 × 10^−4^	6.50 × 10^−4^	2.80 × 10^−4^	7.30 × 10^−3^
	FM	6.70 × 10^−4^	7.30 × 10^−4^	5.10 × 10^−4^	3.90 × 10^−3^	1.70 × 10^−3^	3.79 × 10^−6^	1.50 × 10^−4^	1.00 × 10^−3^	3.30 × 10^−4^	9.00 × 10^−3^

M, male; FM, female.

**Table 6:** Carcinogenic risks of some potentially toxic metals in dry *Crataegus* samples.

**Samples**	**Gender**	**Cr**	**Pb**	**Cd**	**Ni**	**As**
*C. tanacetifolia*	M	1.80 × 10^−6^	1.10 × 10^−10^	4.90 × 10^−9^	3.10 × 10^−5^	2.10 × 10^−7^
	FM	2.10 × 10^−6^	1.30 × 10^−10^	1.50 × 10^−8^	4.80 × 10^−5^	2.50 × 10^−7^
*C. orientalis*	M	2.90 × 10^−6^	1.10 × 10^−10^	4.80 × 10^−9^	1.90 × 10^−5^	2.10 × 10^−7^
	FM	3.40 × 10^−6^	1.30 × 10^−10^	1.50 × 10^−8^	1.90 × 10^−5^	2.50 × 10^−7^
*C. microphylla*	M	5.90 × 10^−6^	1.10 × 10^−10^	4.80 × 10^−9^	2.20 × 10^−5^	1.30 × 10^−7^
	FM	6.90 × 10^−6^	1.30 × 10^−10^	1.50 × 10^−8^	3.40 × 10^−5^	1.50 × 10^−7^

M, male; FM, female.

## Data Availability

Data will be made available on request. Data will be provided by the corresponding author upon request.

## Abbreviations

The following abbreviations are used in this manuscript:

PTE	Potentially Toxic Elements
RDA	Recommended dietary allowance
THQ	Target hazard quotient
EDI	Estimated daily intake
CR	Target cancer risk
HI	Hazard index
TDI	Tolerable daily intake
EFSA	European Food Safety Authority
