# Correction: Şeker et al. Comparative Analysis of Phenolic, Carotenoid, and Elemental Profiles in Three *Crataegus* Species from Şebinkarahisar, Türkiye: Implications for Nutritional Value and Safety. *Molecules* 2025, *30*, 2934

**DOI:** 10.3390/molecules30173647

**Published:** 2025-09-08

**Authors:** Mehmet Emin Şeker, Ayşegül Erdoğan, Emriye Ay

**Affiliations:** 1 School of Tobacco Expertise, Manisa Celal Bayar University, Akhisar 45200, Türkiye; 2 Application and Research Centre for Testing and Analysis (Ege MATAL), Ege University, İzmir 35040, Türkiye; 3 Department of Chemistry, Manisa Celal Bayar University, Yunusemre 45140, Türkiye; emriye.ay@cbu.edu.tr

## Error in Table

In the original publication [1], there was a mistake in “**Table 6.** Carcinogenic risks of some potentially to × ic metals in dry *Crataegus* samples.” as published. Table 5 was re-copied instead of Table 6. The corrected “**Table 6.** Carcinogenic risks of some potentially toxic metals in dry *Crataegus* samples.” appears below (Table 6). The authors state that the scientific conclusions are unaffected. This correction was approved by the Academic Editor. The original publication has also been updated.

## Figures and Tables

**Table 6:** Carcinogenic risks of some potentially toxic metals in dry *Crataegus* samples.

**Samples**	**Gender**	**Cr**	**Pb**	**Cd**	**Ni**	**As**
*C. tanacetifolia*	M	1.80 × 10^−6^	1.10 × 10^−10^	4.90 × 10^−9^	3.10 × 10^−5^	2.10 × 10^−7^
	FM	2.10 × 10^−6^	1.30 × 10^−10^	1.50 × 10^−8^	4.80 × 10^−5^	2.50 × 10^−7^
*C. orientalis*	M	2.90 × 10^−6^	1.10 × 10^−10^	4.80 × 10^−9^	1.90 × 10^−5^	2.10 × 10^−7^
	FM	3.40 × 10^−6^	1.30 × 10^−10^	1.50 × 10^−8^	1.90 × 10^−5^	2.50 × 10^−7^
*C. microphylla*	M	5.90 × 10^−6^	1.10 × 10^−10^	4.80 × 10^−9^	2.20 × 10^−5^	1.30 × 10^−7^
	FM	6.90 × 10^−6^	1.30 × 10^−10^	1.50 × 10^−8^	3.40 × 10^−5^	1.50 × 10^−7^

M, male; FM, female.
